# Selective Killing of Cancer Cells by Nonplanar Aromatic Hydrocarbon‐Induced DNA Damage

**DOI:** 10.1002/advs.201901341

**Published:** 2019-09-16

**Authors:** Yan Zhou, Fuwei Gan, Yuanliang Zhang, Xiaozhen He, Chengshuo Shen, Huibin Qiu, Peifeng Liu

**Affiliations:** ^1^ State Key Laboratory of Oncogenes and Related Genes Shanghai Cancer Institute Ren Ji Hospital School of Medicine Shanghai Jiao Tong University Shanghai 200032 China; ^2^ Central Laboratory Ren Ji Hospital School of Medicine Shanghai Jiao Tong University Shanghai 200127 China; ^3^ School of Chemistry and Chemical Engineering State Key Laboratory of Metal Matrix Composites Shanghai Jiao Tong University Shanghai 200240 China; ^4^ State Key Laboratory of Medical Genomics Shanghai Institute of Hematology Ruijin Hospital School of Medicine Shanghai Jiao Tong University Shanghai 200025 China

**Keywords:** cancer therapy, DNA interstrand crosslinks, helicene, nonplanar, polycyclic aromatic hydrocarbons

## Abstract

A large number of current chemotherapeutic agents prevent the growth of tumors by inhibiting DNA synthesis of cancer cells. It has been found recently that many planar polycyclic aromatic hydrocarbons (PAHs) derivatives, previously known as carcinogenic, display anticancer activity through DNA cross‐linking. However, the practical use of these PAHs is substantially limited by their low therapeutic efficiency and selectivity toward most tumors. Herein, the anticancer property of a nonplanar PAH named [4]helicenium, which exhibits highly selective cytotoxicity toward liver, lung cancer, and leukemia cells compared with normal cells, is reported. Moreover, [4]helicenium effectively inhibits tumor growth in liver cancer‐bearing mice and shows little side effects in normal mice. RNA sequencing and confirmatory results demonstrate that [4]helicenium induces more DNA damage in tumor cells than in normal cells, resulting in tumor cell cycle arrest and apoptosis increment. This study reveals an unexpected role and molecular mechanism for PAHs in selectively killing tumor cells and provides an effective strategy for precision cancer therapies.

## Graphical abstract

**Nonplanar polycyclic aromatic hydrocarbons (PAH) [4]helicenium** selectively kills cancer cells and efficiently inhibits tumor growth, and has no obvious side effect on normal cells. This study provides a distinctive pathway for the use of nonplanar PAHs for the treatment of tumors.

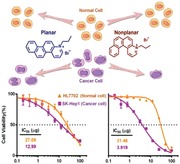

## 1 Introduction

Cancer is a worldwide public health problem and ranks top 2 in leading causes of death in this century.^1^ Recently, new immunotherapy techniques targeting at the immune checkpoint, represented by PD‐1/PD‐L1, exhibit remarkably encouraging therapeutic efficacy and thus have attracted intensive attention.^2^ However, due to the lack of tumor targeting, these drugs still need to be used with conventional chemotherapy.^[2a,3]^ Actually, traditional anticancer medicines remain the primary choices for most patients. Depending on the different mechanisms, the traditional anticancer agents can be classified into several categories: anti‐DNA synthesis and metabolism drugs (e.g., cisplatin,^4^ 5‐FU^5^), protein synthesis inhibitors (e.g., asparaginase^6^), mitotic disruptors (e.g., paclitaxel^7^), etc. Nevertheless, the clinical use of these traditional anticancer medicines is normally limited by their low selectivity, serious side effects, and multidrug resistance.^8^ Therefore, it is imperative to develop advanced cytotoxic drugs with higher curing efficiency and safety.

Polycyclic aromatic hydrocarbons (PAHs) and their derivatives are considered potentially carcinogenic.^9^ For most PAHs, they possess a planar π‐conjugated structure and can interact with DNA by inserting into the base pairs as intercalator or binding to the grooves of DNA due to their hydrophobicity. It is accepted that these chemical compounds can induce tumor and subsequently trigger cellular toxicity‐mediated apoptosis, autophagic cell death, and immune suppression.^10^ However, the derivatives of PAHs after metabolic activation in vivo actually show carcinogenic effects, while PAHs usually would not induce tumors directly.^11^ Besides, based on the similar property of binding to DNA, some PAHs such as substituted anthracene^12^ and pyrene ring systems have exhibited an anticancer efficiency and been also used clinically to treat several leukemias and some solid tumors.^[12a,13]^ Therefore, the biological functions of PAHs derivatives as carcinogens or anticancer agents might highly depend on their molecular skeletons and substitutions.^14^ Compared with planar PAHs, nonplanar PAHs intrinsically own higher solubility in water due to the weaker π–π interaction between two molecules, which make them as better candidates for biological applications, especially as the antitumor drugs.^15^ However, the bioactivities of nonplanar PAHs such as helicenes,^16^ fullerenes,^17^ circulenes,^18^ and corannulenes^19^ are still rarely explored. Herein, we synthesized a nonplanar PAH ([4]helicenium), which possesses good solubility in water and can efficiently bind to DNA. We found that [4]helicenium presented highly selectivity toward hepatocellular carcinoma (HCC), lung cancer, and leukemia cells, and effectively inhibited the growth of liver tumors in tumor‐bearing mice. This study first provides evidence that the conversion of planar PAHs into nonplanar PAHs enhances antitumor capability without significant side effects on normal cells.

## 2 Results

### 2.1 [4]Helicenium Can Bind with DNA by π–π Interaction

**Figure 1:**
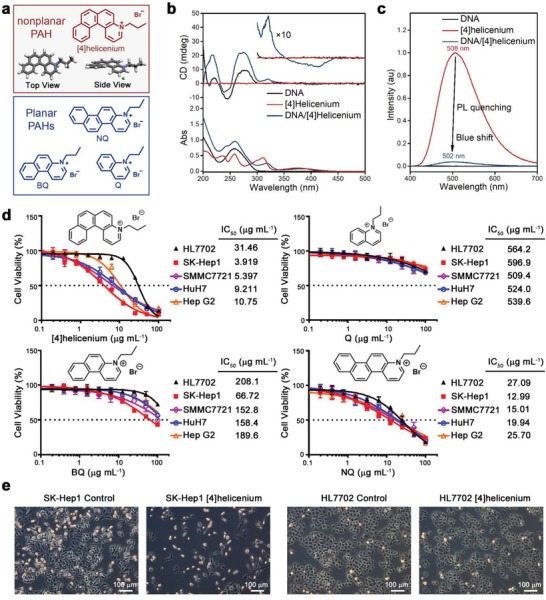
Spectroscopy of [4]helicenium with DNA and in vitro cytotoxicity of PAHs in HCC cells. a) Molecular structure of [4]helicenium, Q, BQ, and NQ. b) UV–vis and CD spectra of DNA, [4]helicenium, and DNA/[4]helicenium complex, *c*_[4]helicenium_ = 200 × 10^−6^ m, *c*_DNA_ = 0.5 mg mL^−1^. c) Photoluminescence (PL) spectra of DNA, [4]helicenium, and DNA/[4]helicenium complex, *c*_[4]helicenium_ = 200 × 10^−6^ m, *c*_DNA_ = 0.5 mg mL^−1^. d) Cell viability analysis of normal hepatocyte HL7702, HCC cells including SK‐Hep1, SMMC7721, HuH7, and Hep G2 treated with [4]helicenium, Q, BQ, and NQ at 0–100 µg mL^−1^ for 24 h. e) Light microscopy images of SK‐Hep1 and HL7702 treated with 10 µg mL^−1^ [4]helicenium. Scale bar represents 100 µm.

Cationic nonplanar PAH derivative [4]helicenium was synthesized via photocyclization and quaternization (**Figure** **1**a up and Figures S1 and S2, Supporting Information). In order to study the interaction between DNA and [4]helicenium, aqueous solutions of [4]helicenium and double‐stranded DNA were mixed to form complexes. UV–vis spectrum showed that upon complexation, the absorption peaks of [4]helicenium at 311 and 373 nm bathchromically moved to 318 and 384 nm, respectively, probably due to the π–π interaction between the base pair of DNA and [4]helicenium. Circular dichroism (CD) spectrum revealed a positive band at 321 nm and a negative band at 414 nm (Figure 1b), indicating an efficient chiral transfer process from DNA to [4]helicenium. Compared with free [4]helicenium, the luminescence intensity of the complex decreased by 96% with a blueshift from 508 to 502 nm, probably as a consequence of static fluorescence quenching with intermolecular nonradiation energy transfer via π–π conjugation (Figure 1c). These results demonstrate that cationic [4]helicenium is in close proximity to DNA and the strong interaction exists between the two components.

### 2.2 [4]Helicenium Selectively Inhibits HCC Cell Growth

The ability of [4]helicenium to bind to DNA in vitro inspired us to evaluate its cytotoxic effects on normal and cancer cells. HCC cells were employed in this work as HCC represents one of the most common tumors with high morbidity and mortality, and more importantly, it lacks effective chemotherapeutic drugs. Aqueous solutions of [4]helicenium at different concentrations (range from 0 to 100 µg mL^−1^) were employed to treat normal hepatocyte cell line HL7702 and four HCC cell lines including SK‐Hep1, SMMC7721, HuH7, and Hep G2. The cell viability was detected by Cell Counting Kit‐8 (CCK8). The viability of HL7702 cell remained almost constant (>88%) with the treatment of [4]helicenium below 12.5 µg mL^−1^ and significantly dropped at higher [4]helicenium concentrations. On the contrary, for HCC cells, their viability started to decrease gradually at a much low [4]helicenium concentration (<1 µg mL^−1^). IC50 (half maximal inhibitory concentration), a common measure of drug effectiveness, was calculated to quantify the cytotoxicity of [4]helicenium. Interestingly, [4]helicenium showed a provocative effect with higher toxicity to HCC cells (IC50_SK‐Hep1_: 3.919 µg mL^−1^, IC50_SMMC7721_: 5.397 µg mL^−1^, IC50_Huh7_: 9.211 µg mL^−1^, IC50_Hep G2_: 10.75 µg mL^−1^) than that to normal hepatocyte HL7702 (IC50: 31.46 µg mL^−1^) (Figure 1d). Planar cationic PAH derivatives, which shared same chemical elements as [4]helicenium but with different aromatic ring numbers, including quinolinium (Q),^20^ benzo[*f*]quinolinium (BQ)^20^ and naphtho[2,1‐*f*]quinolinium (NQ), were also introduced for comparison (Figure 1a bottom and Figures S3–S8, Supporting Information). With Q and NQ treatment, the CCK8 curves of normal hepatocyte HL7702 and HCC cells appeared to overlap with each other. In the case of BQ, the CCK8 curves of HL7702 cell slightly violated from others above 10 µg mL^−1^ (Figure 1d). The IC50 of these planar PAHs toward HCC cells (IC50(Q): 509.4–596.9 µg mL^−1^, IC50(BQ): 66.72–189.6 µg mL^−1^, IC50(NQ): 12.99–25.70 µg mL^−1^) decreased accompanied by the increasing number of aromatic rings, but displayed no significant selectivity compared with that toward normal hepatocyte HL7702 (IC50(Q): 564.2 µg mL^−1^, IC50(BQ): 208.1 µg mL^−1^, IC50(NQ): 27.09 µg mL^−1^) (Figure 1d). Moreover, the captured cell morphology further confirmed that [4]helicenium with the concentration of 10 µg mL^−1^ showed stronger cytotoxic effects on HCC cell SK‐Hep1 as the cell number in the fields was significantly reduced, while [4]helicenium treatment had no effect on the cellular morphology and cell number of normal hepatocyte HL7702 (Figure 1e). Apparently, [4]helicenium possesses a much higher selectivity than the planar PAH analogs to kill HCC cells.

### 2.3 [4]Helicenium Promotes Cell Apoptosis and Cell Cycle Arrest in HCC Cell

**Figure 2:**
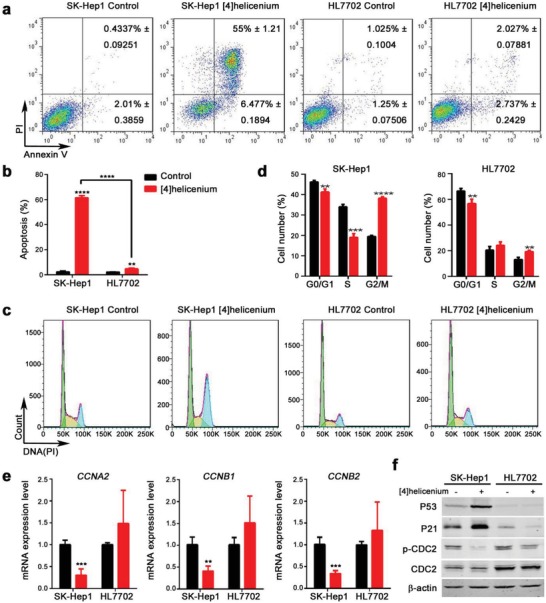
In vitro cell apoptosis and cell cycle analysis in SK‐Hep1 and HL7702 cells. SK‐Hep1 and HL7702 were treated with 10 µg mL^−1^ [4]helicenium for 24 h. a,b) Flow cytometry analysis of apoptotic cell population of SK‐Hep1 and HL7702 before and after [4]helicenium treatment by Annexin V/PI staining. c,d) Flow cytometry analysis of cell cycle of SK‐Hep1 and HL7702 before and after [4]helicenium treatment by PI staining. e) Q‐PCR analysis of cyclin‐related genes (*CCNA2*, *CCNB1*, *CCNB2*) expressed in SK‐Hep1 and HL7702 before and after [4]helicenium treatment. GAPDH was used as an internal control. f) Western blot analysis of the protein expression levels of P53, P21, p‐CDC2, and CDC2 in SK‐Hep1 and HL7702 before and after [4]helicenium treatment. β‐actin was used as a loading control. Error bars represent mean ± S.D. ** *p* < 0.01, *** *p* < 0.001, **** *p* < 0.0001 (Student's *t*‐test).

Low cell viability was often attributed to the increase of cell apoptosis and/or cell cycle arrest. Hence, flow cytometry analysis (FACS) was performed to detect the states of apoptosis and cell cycle. [4]Helicenium significantly increased the proportion of late apoptotic cells (Annexin V^+^ PI^+^ (propidium iodide)) in SK‐Hep1 (55% ± 1.21) compared to HL7702 (2.027% ± 0.07881) (*p* < 0.0001), and also appropriately increased the proportion of early apoptotic cells (Annexin V^+^ PI^−^) (SK‐Hep1 6.477% ± 0.1894 vs HL7702 2.737% ± 0.2429, *p* = 0.003), implying that the increased level of cell apoptosis was responsible for the killing effects of [4]helicenium (**Figure** **2**a,b). Moreover, PI, a fluorescent intercalator that can linearly bind to DNA without sequence preference, was used to analyze the cell cycle states of different DNA content by calculating PI fluorescence intensity. PI staining of these cells expounded that [4]helicenium significantly arrested cell cycle in G2/M phase (19.51% ± 0.35 vs 38.24% ± 0.41, *p* < 0.0001) of SK‐Hep1, accompanied by a decrease in the proportion of G0/G1 (46.22% ± 0.44 vs 41.30% ± 0.88, *p* = 0.0076) and S (34.04% ± 0.67 vs 19.12% ± 1.042, *p* = 0.0003) phase cells. Whereas the proportion of G2/M phase (13.22% ± 1.02 vs 19.40% ± 0.59, *p* = 0.0064), as well as the G0/G1 phase (66.54% ± 1.14 vs 55.76% ± 1.93, *p* = 0.012), were slightly blocked in HL7702 after [4]helicenium treatment, while the proportion of S phase (20.48% ± 1.61 vs 24.25% ± 1.57, *p* = 0.17) increased slightly (although there was no statistical difference) (Figure 2c,d), indicating that the stronger cell cycle arrest caused by [4]helicenium may account for its antitumor selectivity. To further validate the variation in the cell cycle, we applied quantitative real‐time PCR (q‐PCR) to quantify the expression of cyclin‐related genes, the accelerator of cell cycle progression. [4]Helicenium significantly inhibited the expression of cyclin‐related genes including *CCNA2* (Fold change (FC) = 0.3, *p* = 0.0002), *CCNB1* (FC = 0.4, *p* = 0.0014), and *CCNB2* (FC = 0.34, *p* = 0.0003) in SK‐Hep1 (Figure 2e). Since the P53‐P21 pathway is the most common brake system of the cell cycle and can be activated by many drugs that lead to cell cycle arrest,^21^ Western blotting was performed to verify the activation of P53‐P21 pathway. The results showed that [4]helicenium significantly activated P53 and P21 in SK‐Hep1, but had no effect on HL7702 (Figure 2f). In addition, p‐CDC2, an important regulator in promoting G2/M phase transition,^22^ was inhibited in SK‐Hep1 after the treatment of [4]helicenium (Figure 2f), further implying that [4]helicenium‐treated HCC cell confronted with a problem of cell cycle progression defects. It appeared that the selective inhibition of HCC cell growth by [4]helicenium is mainly due to cell cycle arrest and cell apoptosis increment.

### 2.4 [4]Helicenium Inhibits Tumor Growth in SK‐Hep1 Tumor‐Bearing Mice In Vivo

Although the selective antitumor ability has been demonstrated in several HCC cells in vitro, it remains unclear whether [4]helicenium has similar effects in vivo. Before performing in vivo experiments in mice, we evaluated the ADMET parameters such as absorption, distribution, metabolism, and toxicity by liquid chromatography‐tandem mass spectrometry (LC‐MS). First, we used human and rat liver microsomes as in vitro models to evaluate the metabolic stability of [4]helicenium. The residual rate of [4]helicenium after incubation for 60 min was 40.9% in human liver microsomes, while the residual rates in rat liver microsomes after 5 and 15 min were 31.4 and 2.6%, respectively (Figure S9a, Supporting Information). The intrinsic clearance (CL_int_) values of human and rat were 37.4 and 876 mL min^−1^ kg^−1^, and the liver clearance (CL_hb_) values of human and rat were 13.3 and 51.9 mL min^−1^ kg^−1^, respectively. These data indicated that [4]helicenium was a medium‐clearing drug in the human body and a high‐clearing drug in rats. Second, the distribution of [4]helicenium in rat tissues and plasma were detected at 1 and 8 h after intravenous injection of 2 mg kg^−1^ [4]helicenium. [4]Helicenium was widely distributed in the tissues examined, distributed mostly in the heart, and secondly distributed in tissues such as kidney, pancreas, uterus, small intestine, and lung. The concentration in the liver was low, consistent with the intrinsic clearance assay, indicating that the liver metabolism of [4]helicenium was very rapid. [4]Helicenium was eliminated more slowly in the rat heart and pancreas. The whole blood‐plasma concentration average distribution ratio of [4]helicenium was about 1.5, indicating that the amount of drug distributed to the red blood cells was greater than the amount remaining in the plasma. The concentration of [4]helicenium in the rat brain was very low, indicating that [4]helicenium hardly crossed the blood–brain barrier (Table S1, Supporting Information). Third, the concentration of [4]helicenium in plasma was measured by LC‐MS/MS at different times after intravenous and intragastric administration in rats. After intravenous injection of 2 mg kg^−1^ [4]helicenium, the concentration of [4]helicenium decreased rapidly, but with a longer terminal elimination half‐life and *t*_1/2_ was 11.7 h (Figure S9b, Supporting Information), which may due to the slow release of [4]helicenium in tissues and red blood cells. [4]Helicenium was difficult to be absorbed after intragastric administration in rats (Figure S9b, Supporting Information). The absolute bioavailability of the rats after a single gavage of 5 mg kg^−1^ [4]helicenium was 1.57%, suggesting that the oral bioavailability is extremely low. Finally, to evaluate the toxicity of [4]helicenium in vivo, we measured the body weight change in four groups of healthy nude mice treated with normal saline, BQ, NQ, and [4]helicenium at a concentration of 3 mg kg^−1^ every other day for four weeks. The body weight profiles showed that BQ, NQ, and [4]helicenium had no significant effects on mouse body weight (Figure S9c, Supporting Information).

**Figure 3:**
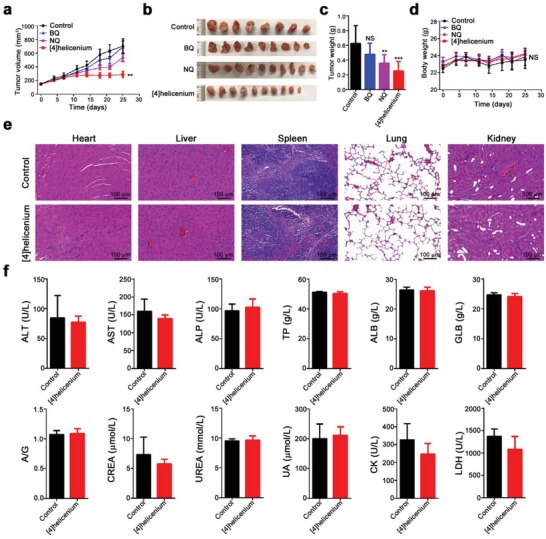
In vivo antitumor efficacy of [4]helicenium on the HCC nude mouse model. a) Tumor growth profiles of SK‐Hep1 tumor‐bearing nude mice treated with normal saline, BQ, NQ, and [4]helicenium. b,c) Photographs and weight of the tumors extracted from the four groups of mice at the end of the experiments. d) Body weight profiles of SK‐Hep1 tumor‐bearing nude mice treated with normal saline, BQ, NQ, and [4]helicenium. e) HE staining of the major organs (i.e., heart, liver, spleen, lung, and kidney) of SK‐Hep1 tumor‐bearing nude mice treated with normal saline and [4]helicenium. f) Liver, kidney, and heart functions of SK‐Hep1 tumor‐bearing nude mice treated with normal saline and [4]helicenium. Error bars represent mean ± S.D. NS: not significant; ** *p* < 0.01, *** *p* < 0.001 (Student's *t*‐test).

The xenograft mouse model is often employed to observe tumorigenesis and access the antitumor effects of small molecular compounds. Two weeks after inoculation of SK‐Hep1 under the dorsal skin of nude mice, tumor‐bearing mice were randomly separated into four groups with an average of the tumor volume of 150 mm^3^ and then intravenously injected with normal saline, BQ, NQ, and [4]helicenium at a concentration of 3 mg kg^−1^ every other day for four weeks. The tumor volume and body weight were monitored during the animal studies and the mice were sacrificed at the endpoint of the antitumor experiments for curative effect observation. Although the volume of HCC tumors continued to grow slowly from 149 to 243 mm^3^ (*p* = 0.0017) during the first three [4]helicenium treatments, since then, [4]helicenium obviously inhibited tumor growth at endpoint (288.6 ± 45.85 mm^3^), while the volume of saline tumors increased nearly fivefold (150.2 ± 15.82 mm^3^ vs 703.9 ± 108.3 mm^3^, *p* < 0.0001) (**Figure** **3**a). However, BQ showed negligible influence on the tumor growth (150.9 ± 14.11 mm^3^ vs 692.1 ± 86.19 mm^3^, *p* < 0.0001) and NQ only had slight therapeutic efficacy on tumors (151.9 ± 11.56 mm^3^ vs 535.4 ± 54.88 mm^3^, *p* < 0.0001) (Figure 3a). Subsequently, tumors were isolated for morphological and gravimetric analysis, and the results uncovered that [4]helicenium inhibited HCC tumor growth more pronounced than the other three groups ([4]helicenium: 0.253 ± 0.04 g vs saline: 0.626 ± 0.085 g, *p* = 0.007) (Figure 3b,c). Noteworthy that the body weight of the four groups of tumor‐bearing mice showed negligible change during the entire observation period (Figure 3d), suggesting that these PAHs had no obvious side effects. To further clarify the safety of [4]helicenium, we collected major organs (i.e., heart, liver, spleen, lung, and kidney) from tumor‐bearing nude mice treated with saline and [4]helicenium for histopathological analysis, and histological examinations demonstrated s [4]helicenium had no significant damage to major organs, such as tissue structure disorder, hemorrhage, or edema (Figure 3e), Moreover, serological indexes indicated that the function of liver, kidney, and heart are normal in [4]helicenium‐treated mice (Figure 3f). These results unequivocally suggest that [4]helicenium can not only kill cancer cells but also show nearly no toxic side effects on normal cells and organs, highlighting that [4]helicenium has the potential application in tumor chemotherapy.

### 2.5 [4]Helicenium Inhibits DNA Replication in HCC Cell

**Figure 4:**
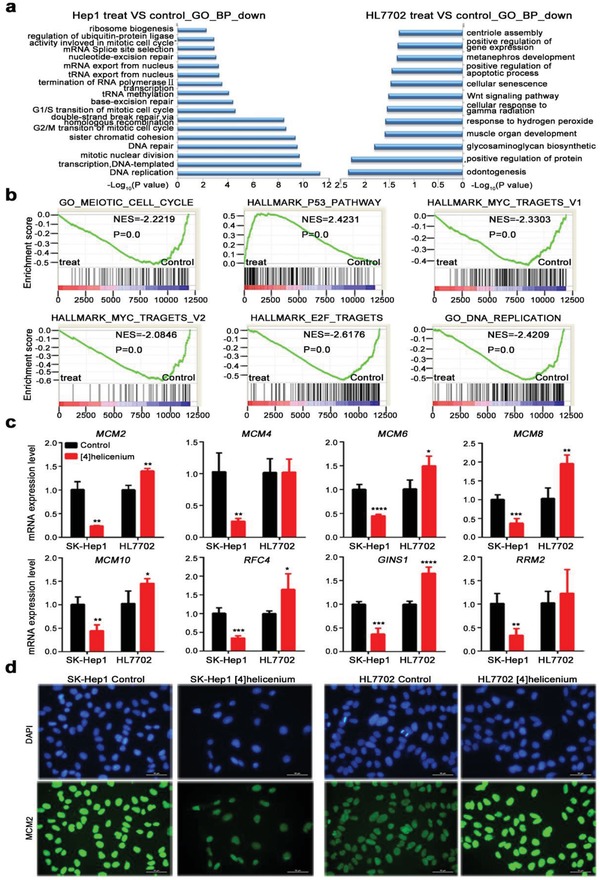
Inhibition of [4]helicenium on DNA replication in SK‐Hep1. a) GO enrichment analysis of downregulated genes in SK‐Hep1 and HL7702 before and after [4]helicenium treatment. b) GSEA of the entire transcriptome of SK‐Hep1 before and after [4]helicenium treatment. c) Q‐PCR analysis of DNA replication‐related genes expressed in SK‐Hep1 and HL7702 before and after [4]helicenium treatment. d) MCM2 immunofluorescence staining of SK‐Hep1 and HL7702 before and after [4]helicenium treatment. Error bars represent mean ± S.D. * *p* < 0.05,** *p* < 0.01, *** *p* < 0.001, **** *p* < 0.0001 (Student's *t*‐test).

To gain deeper insights into the underlying molecular mechanism of selective killing of HCC cells by [4]helicenium, RNA sequencing analysis (RNA‐seq) was performed. The results showed that 1229 genes were abnormally expressed in HL7702 after [4]helicenium treatment, including 350 downregulated and 879 upregulated genes (FC ≤ 0.5 or FC ≥ 2; *p* < 0.05). In contrast, in HCC cell SK‐Hep1, up to 6964 genes were changed after the treatment of [4]helicenium, of which 1875 genes were downregulated and 5089 genes were upregulated, indicating that [4]helicenium can remodel SK‐Hep1 transcriptome. *CCNA2* (FC = 0.17, *p* < 0.0001), *CCNB1* (FC = 0.43, *p* < 0.0001), and *CCNB2* (FC = 0.38, *p* < 0.0001) were all downregulated after [4]helicenium treatment in RNA‐seq, which were consistent with q‐PCR results (Figure 2e). Gene ontology (GO) analysis of differential genes in SK‐Hep1 showed that the downregulated genes were mainly enriched in the mitotic nuclear division, cell cycle regulation (G2/M and G1/S transition), DNA replication, and DNA repair (homologous recombination (HR) repair, base‐excision repair, and nucleotide‐excision repair). In addition, multiple RNA‐related biological processes are found to be significantly enriched, such as mRNA transcription, splicing, and mRNA/tRNA export from the nucleus (**Figure** **4**a left). These changes in biological processes are a good explanation for the observed phenotype (i.e., cell cycle arrest) of growth inhibition of HCC cells. However, normal hepatocyte did not show enrichment of these biological processes after [4]helicenium treatment (Figure 4a right), further suggesting that SK‐Hep1 and HL7702 respond differently to [4]helicenium.

Next, Gene Set Enrichment Analysis (GSEA), a global view of the transcriptome profile, was also performed in SK‐Hep1. In line with the lower accelerator and higher brake of cell cycle activity mentioned above (Figure 2), GSEA revealed the downregulation of the meiotic cell cycle (normalize enrichment score, NES = −2.2219, *p* = 0.00) and upregulation of P53 pathway (NES = 2.4231, *p* = 0.00) in SK‐Hep1 treated with [4]helicenium (Figure 4b). Besides the inhibitor of cell proliferation (e.g., P53), MYC and E2F, two positive regulators of cell growth, were also significantly attenuated in SK‐Hep1 treated with [4]helicenium (Figure 4b). Furthermore, it should be noted that DNA replication was significantly inhibited by [4]helicenium in SK‐Hep1 (NES = −2.4209, *p* = 0.00) (Figure 4b). However, [4]helicenium‐treated HL7702 displayed a different transcriptional pattern by GSEA (Figure S10a, Supporting Information). P53 pathway (NES = 1.1, *p* = 0.287) and DNA replication (NES = 0.9, *p* = 0.7) did not show significant change in [4]helicenium‐treated HL7702. Similar to SK‐Hep1, [4]helicenium also can inhibit MYC target gene sets in HL7702, but the heatmap of the individual gene expression of MYC target gene sets indicated that downregulation of these genes was stronger in SK‐Hep1 than in HL7702 (Figure S10b,c, Supporting Information). The aforementioned cell cycle revealed that [4]helicenium arrested SK‐Hep1 and HL7702 at G2/M phase (Figure 2d), but in contrast to SK‐Hep1, meiotic cell cycle (NES = 1.55, *p* = 0.004) was positive enrichment in HL7702 (Figure S10a,d, Supporting Information), indicating that G2/M cell cycle arrest may play a different role in HL7702 and SK‐Hep1 after [4]helicenium treatment. E2F targets (NES = 1.23, *p* = 0.107) also displayed a similar change like meiotic cell cycle despite no statistical significance (Figure S10a, Supporting Information). And the heatmap of the individual gene showed that half of the E2F targets were upregulated in HL7702 but most of them were downregulated in SK‐Hep1 after [4]helicenium treatment (Figure S10e, Supporting Information). Considering that [4]helicenium can bind to DNA in vitro (Figure 1b,c), the notion that [4]helicenium firstly induce DNA replication defects, and eventually arrest HCC cell cycle by binding to DNA in vivo can be supported. Subsequently, q‐PCR was used to verify the changes of DNA replication‐related genes, and the results showed that multiple minichromosome maintenance complex (MCM) genes, including *MCM2* (FC = 0.24 ± 0.006, *p* = 0.0014), *MCM4* (FC = 0.25 ± 0.022, *p* = 0.0021), *MCM6* (FC = 0.45 ± 0.014, *p* < 0.0001), *MCM8* (FC = 0.37 ± 0.06, *p* = 0.0004), *MCM10* (FC = 0.44 ± 0.06, *p* = 0.0014), *RFC4* (FC = 0.35 ± 0.026, *p* = 0.0002), *GINS1* (FC = 0.37 ± 0.06, *p* = 0.0001), and *RRM2* (FC = 0.33 ± 0.072, *p* = 0.0018), were downregulated in SK‐Hep1 treated with [4]helicenium (Figure 4c). Further immunofluorescence (IF) investigation manifested that [4]helicenium significantly inhibited the expression of MCM2 protein in SK‐Hep1 (Figure 4d). In contrast, some of MCM genes (e.g., *MCM2*, *MCM8*, *MCM10*, *GINS1*) displayed a slight upregulation in [4]helicenium‐treated HL7702 (Figure 4c), and the fluorescence intensity of MCM2 was also augment (Figure 4d), which was in parallel with the slight increase in S phase of cell cycle (Figure 2d). Collectively, integrating these data denote that the distinction in response to DNA replication stress induced by [4]helicenium, which is collapsed in HCC cells but compensated in normal cells, are mainly responsible for compound selectivity.

### 2.6 [4]Helicenium Causes DNA Strand Cross‐Linking in HCC Cell

**Figure 5:**
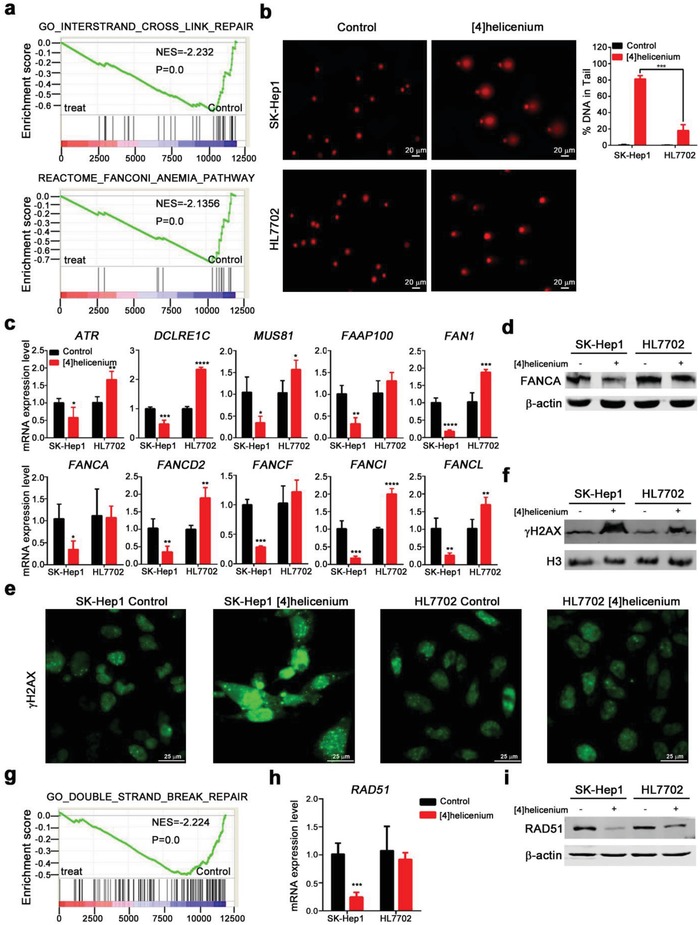
Inhibition of [4]helicenium on DNA ICLs repair and DSBs repair in SK‐Hep1. a) GSEA of ICLs repair and FA signaling pathway of SK‐Hep1 before and after [4]helicenium treatment. b) Comet assay images (right) and statistical results (left) of SK‐Hep1 and HL7702 before and after [4]helicenium treatment. c) Q‐PCR analysis of ICLs repair and FA signaling pathway‐related genes expressed in SK‐Hep1 and HL7702 before and after [4]helicenium treatment. d) Western blot analysis of the protein expression level of FANCA in SK‐Hep1 and HL7702 before and after [4]helicenium treatment. e) γH2AX immunofluorescence staining of SK‐Hep1 and HL7702 before and after [4]helicenium treatment. f) Western blot analysis of the protein expression level of γH2AX in SK‐Hep1 and HL7702 before and after [4]helicenium treatment. Histone H3 was used as a loading control. g) GSEA of DSBs repair of SK‐Hep1 before and after [4]helicenium treatment. h) Q‐PCR analysis of *RAD51* expressed in SK‐Hep1 and HL7702 before and after [4]helicenium treatment. i) Western blot analysis of the protein expression level of RAD51 in SK‐Hep1 and HL7702 before and after [4]helicenium treatment. Error bars represent mean ± S.D. * *p* < 0.05,** *p* < 0.01, *** *p* < 0.001, **** *p* < 0.0001 (Student's *t*‐test).

Given that several mechanisms are responsible for the anti‐DNA syntheses, such as folic acid antagonist for the inhibition of purines and thymidylates, pyrimidine and purine analogs for incorporating into DNA, and platinum for DNA crosslink, we attempted to detect the mechanism of action for [4]helicenium. Since [4]helicenium can bind to DNA, the mechanism by which [4]helicenium inhibits cancer cell growth may be similar to platinum,^23^ which cause intracellular DNA interstrand crosslinks (ICLs). Indeed, a set of ICLs was negatively enriched in SK‐Hep1 by GSEA analysis (NES = −2.232, *p* = 0.00) (**Figure** **5**a up). Next, the comet assay (an effective ICLs detection method) elucidated that [4]helicenium rendered serious cross‐linkage damage of DNA strands in SK‐Hep1 (81.29% ± 2.375) than in HL7702 (18.12% ± 4.081) (*p* = 0.0002) (Figure 5b). It is well known that ICLs are the most serious form of DNA damage, not only destroying the structure and function of DNA but also inhibiting DNA synthesis. In the cellular circumstance, Fanconi anemia (FA) pathway can participate in the repair of ICLs in vivo.^24^ However, the FA pathway was severely damaged in SK‐Hep1 after [4]helicenium treatment confirmed by GSEA (NES = −2.1356, *p* = 0.00) (Figure 5a bottom). Meanwhile, q‐PCR of FA‐related genes (e.g., *FANCA*, FC = 0.35 ± 0.095, *p* = 0.0109) and Western blotting of FANCA further established this opinion that [4]helicenium attenuated FA repair in SK‐Hep1 (Figure 5c,d). Interestingly, consistent with cell cycle and DNA replication response in HL7702 treated with [4]helicenium, many FA repair‐related genes expression (e.g., *DCLRE1C*, *FAN1*, *FANCD2*, *FANCI*, *FANCL*) were upregulated (Figure 5c). To better understand whether different DNA damage response (DDR) in normal and tumor cells was [4]helicenium dose‐dependent, HL7702 and SK‐Hep1 were treated with [4]helicenium at a concentration ranging from 2 to 40 µg mL^−1^, and then the transcriptional level of the DDR genes, including DNA replication‐ and FA‐related genes, were quantified by q‐PCR. [4]Helicenium above 2 µg mL^−1^ dramatically attenuated DDR‐related genes (e.g., *MCM2, MCM4, MCM6, MCM8, MCM10, RFC4, RRM2, GINS1, FAAP100, RAN1, FANCA, FANCD2, FANCF, FANCI, FANCL*) expression in SK‐Hep1 (Figure S11a, Supporting Information). However, only 20 or 40 µg mL^−1^ of [4]helicenium inhibited the expression level of DDR‐related genes in HL7702, and [4]helicenium below 10 µg mL^−1^ significantly upregulated these genes expression in HL7702, indicating a stress‐compensatory response to DNA damage induced by low‐dose [4]helicenium (Figure S11b, Supporting Information). These data suggested that HL7702 and SK‐Hep1 had a different DDR caused by [4]helicenium in the appropriate therapeutic window.

Based on the severity of ICLs, if the ICLs injury cannot be repaired in time, or the ICLs damage exceeds the repair capacity of the cells themselves, these will cause serious DNA double‐strand breaks (DSBs), leading to prevent DNA replication and transcription.^25^ As phospho‐histone H2A.X (γ H2AX) is a hallmark of DSBs,^26^ its expression level was detected to evaluate the effects of [4]helicenium on DNA DSBs. The distribution of γH2AX in cells was detected by IF, and the results showed a significant increase in the intensity of γ H2AX in [4]helicenium‐treated SK‐Hep1 (Figure 5e). The Western blotting results also confirmed the increase of γ H2AX level in SK‐Hep1 (Figure 5f), while only a slight increase in the expression level of γ H2AX in HL7702, suggesting that [4]helicenium can cause extensive DNA DSBs in HCC cells. Given that DSBs can be repaired by DNA double‐strand recombination, yet the increased level of γ H2AX in [4]helicenium‐treated SK‐Hep1 may imply that this DSBs repair was also blocked. The transcriptome data showed that DNA double‐stranded recombination repair was significantly inhibited (NES = −2.224, *p* = 0.0) (Figure 5g), and the results of q‐PCR and Western blotting also revealed that the expression level of *RAD51*, an important gene for DNA HR repair,^27^ was reduced in [4]helicenium‐treated SK‐Hep1 (FC = 0.34 ± 0.053, *p* = 0.0002) (Figure 5h,i). These results suggest that inducing DNA ICLs is the major role of [4]helicenium in HCC cells, then leading to fatal DNA DSBs and cell death. This cytostatic medication of [4]helicenium is similar to the currently widely used platinum chemotherapeutic drugs such as cisplatin. However, the clinical application of cisplatin is often limited by its low selectivity between normal cells and cancer cells and drug resistance. In this study, we found that cisplatin could not effectively kill HCC cell SK‐Hep1, and it even behaved a stronger cytotoxic effect on normal hepatocyte HL7702 (Figure S12, Supporting Information). This result suggested that [4]helicenium may have a broader clinical application than cisplatin. In addition to DDR and DNA repair mechanisms that allow [4]helicenium to selectively kill tumor cells, we speculated that the distribution of [4]helicenium inside and outside the cell may be involved in selectivity. The results of LC‐MS analysis exhibited that the intracellular concentration of [4]helicenium was much higher in SK‐Hep1 than in HL7702, but extracellular concentration showed the opposite result (Figure S13, Supporting Information). Taken together, these results indicated that S phase‐dominant SK‐Hep1 cell absorbed more [4]helicenium so that caused more severe DNA damage and inhibited the transcription of DNA repair‐related genes, ultimately led to cell cycle arrest and apoptosis in SK‐Hep1.

### 2.7 [4]Helicenium Can Also Selectively Inhibit Lung Cancer and Leukemia Cell Growth

**Figure 6:**
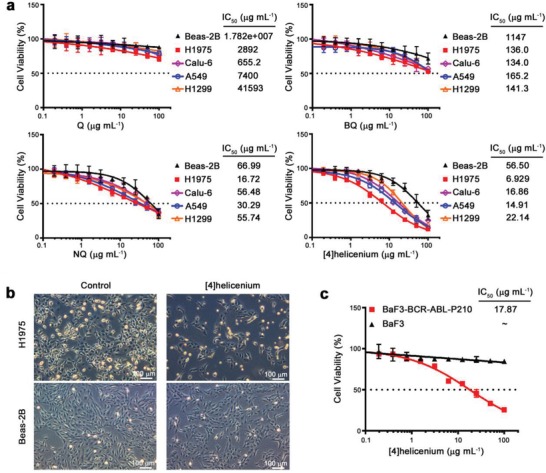
In vitro cytotoxicity of PAHs in lung cancer cells. a) Cell viability analysis of normal lung epithelial cell Beas‐2B and lung cancer cell including H1975, Calu‐6, A549, and H1299 treated with Q, BQ, NQ, and [4]helicenium at 0–100 µg mL^−1^ for 24 h. b) Light microscopy images of H1975 and Beas‐2B treated with 10 µg mL^−1^ [4]helicenium. c) Cell viability analysis of proB cell line BaF3 and leukemia cell line BaF3‐BCR‐ABL‐P210 treated with [4]helicenium at 0–100 µg mL^−1^.

To address whether the selective antitumor ability of [4]helicenium is HCC specific or applicable to other tumors, normal lung epithelial cell Beas‐2B and lung cancer cells including H1975, Calu‐6, A549, and H1299 were treated with [4]helicenium using the same treatment strategy as HCC cells mentioned above. CCK‐8 assays demonstrated that [4]helicenium can also selectively kill lung cancer cells, and planar PAHs (Q, BQ, NQ) displayed antitumor effects with aromatic ring number‐dependent cytotoxicity on lung cancer cells which are similar to HCC cells (**Figure** **6**a). The cell morphology also confirmed the stronger cytotoxic effects of [4]helicenium on lung cancer cells compared with those on normal cells (Figure 6b). In addition, proB cell BaF3 and leukemia cell BaF‐BCR‐ABL‐P210, a well‐known system used to evaluate differences in normal and leukemia cells with the same genetic background,^28^ were treated with [4]helicenium. Consistent with HCC and lung cancer cells, [4]helicenium displayed a high selectivity on leukemia cell relative to parental BaF3 cell (Figure 6c). These results suggest that [4]helicenium can not only show high antitumor activity and selectivity in HCC cells but also be effective in other cancer cells.

## 3 Discussion

PAHs including benzo[a]pyrene, anthracene, and dioxin are widespread in nature and are commonly considered as environmental pollutants which would potentially induce tumors. Actually, most PAHs would not induce cancer directly unless transforming to biologically active molecules after metabolism.^11^ Besides, as DNA intercalators or DNA binders, PAHs share similar mechanism with traditional chemotherapeutic drugs which can inhibit biological functions of DNA and induce apoptosis. This feature means that PAHs can constitute a potential category for novel antitumor agents. And the structures of molecules might be crucial for antitumor activities and selectivities.

**Figure 7:**
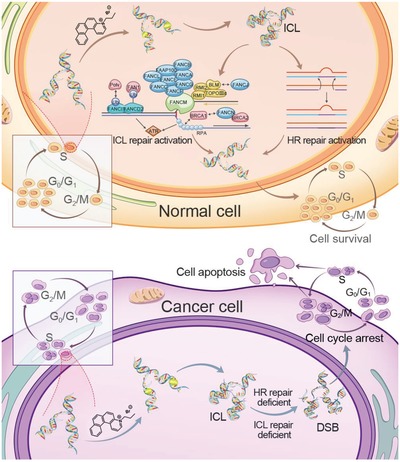
Molecular pattern of selective killing of cancer cells by [4]helicenium. In normal cells, [4]helicenium binds to the DNA of the S phase cells to induce ICLs, which triggers FA and HR repair to remove DNA lesions and then maintains normal cell cycle. In cancer cells, [4]helicenium leads to cell cycle arrest and apoptosis due to the lack of ability to repair ICLs‐related DNA damage.

In this study, our results highlighted that [4]helicenium, a nonplanar PAH, preferentially eradicated cancer cells, and also unearthed rudimentary molecular mechanism of selective‐killing to the tumor. [4]Helicenium can bind directly to the DNA in both normal and cancer cells, however, different cell cycle states and endogenous DNA damage response led to selective tumor killing by [4]helicenium. On the one hand, nearly half of the cancer cells were in the S phase, enabling cancer cells to interact with [4]helicenium easier than normal cells. On the other hand, DNA ICLs and DSBs damages which were induced by [4]helicenium triggered endogenous DNA repair including FA and HR repair in normal cells. In contrast, cancer cells cannot effectively initiate DNA damage response and DNA repair. As a consequence, cell cycle arrest and apoptosis were activated to eradicate unrepaired cancer cells (**Figure** **7**). ICLs can cause serious DNA damage, and some ICL‐inducing agents including platinum drugs are still the most widely used clinical chemotherapy agents, leading to inhibition of tumor DNA synthesis. However, the chemoresistance of platinum drugs remains a big issue in clinical applications. We found that [4]helicenium also killed tumor cells by inducing DNA crosslinks, which encouraged us to evaluate the antitumor activity of [4]helicenium in cisplatin‐resistant cancer cells. Our data showed that cisplatin‐resistant SK‐Hep1 was still sensitive to [4]helicenium, indicating that [4]helicenium induced a different DNA cross‐linking from cisplatin to kill tumor cells. Hence, [4]helicenium will bring new hope for cisplatin‐resistant patients in future clinical applications.

## 4 Conclusion

In conclusion, we have explored the biological activity of nonplanar PAH [4]helicenium and demonstrated that [4]helicenium selectively kills cancer cells and inhibits tumor growth in liver cancer‐bearing mice without obvious toxic side effects on normal cells and mice. Our data suggest that differences in cell cycle states, biased use of DDR and therapeutic window contribute to the selectivity of [4]helicenium between normal and tumor cells. Furthermore, our study also suggests that [4]helicenium can be used as broad‐spectrum anticancer agents for many other highly proliferative tumors, such as leukemias. Additionally, we are currently investigating various other nonplanar PAHs with more aromatic ring numbers and their chiral characteristics to further enhance the tumor therapeutic efficiency and selectivity.

## 5 Experimental Section

*Synthesis and Characterization of PAHs*: Most experiments were performed under an atmosphere of dry nitrogen using standard Schlenk techniques. Commercially available reagents were used as received without further purification. Naphtho[2,1‐*f*  ]quinoline and 4‐aza[4]helicene were synthesized according to the previous literature.^29^ The structure of PAHs was characterized by NMR spectra, which were recorded on a Bruker AVANCE III HD 500 MHz spectrometer. ^1^H and ^13^C chemical shifts were determined using residual signals of the deuterated solvents or using TMS as the internal standard, and the reported in parts per million (ppm) relative to TMS.

*Complexation and Spectral Studies*: DNA with average base pair number of 2000 was purchased from Sigma‐Aldrich. For complexation, DNA and cationic PAHs were dissolved in deionized water with the concentration of 1.6 and 0.4 × 10^−3^ m, respectively, then two aqueous solutions were mixed together with the volume ratio of 1:1. The solutions of DNA/PAHs complexes were characterized by UV–visible (UV–vis), CD, and fluorescence spectra. The UV–vis absorption spectra were recorded on a Shimadzu UV‐2600 spectrometer at 20 °C in a 1 mm quartz cell. Electronic circular dichroism (ECD) spectra were measured on a Jasco J‐815 spectropolarimeter at 20 °C in a 1 mm quartz cell. Fluorescence spectra and quantum yield were measured on a Horiba FL‐3 fluorescence spectrometer (Horiba, Japan).

*Cell Culture and Animals*: Human hepatocellular carcinoma cell line SK‐Hep1, SMMC7721, HuH7, and Hep G2, hepatocyte HL7702, lung epithelial cell Beas‐2B, lung cancer cell line H1975, Calu‐6, A549, and H1299 were purchased from the Cell Bank of the Type Culture Collection of Chinese Academy of Sciences (Shanghai, China). Mouse proB cell line BaF3 and chronic myeloid leukemia (CML)‐like leukemia cell line BaF3‐BCR‐ABL‐P210 were provided by the Shanghai Institute of Hematology. SK‐Hep1, HuH7, Hep G2, Calu‐6, and Beas‐2B were cultured in Dulbecco's modified Eagle medium (DMEM) (GIBCO, Thermo Fisher Scientific) supplemented with 10% fetal bovine serum (FBS) (Gibco, Thermo Fisher Scientific). HL7702, H1975 H1299, and BaF3‐BCR‐ABL‐P210 were cultured in Roswell Park Memorial Institute (RPMI) 1640 medium (GIBCO, Thermo Fisher Scientific) with 10% FBS. BaF3 was cultured in 1640 medium with 10% FBS and 10% mouse interleukin‐3 (IL‐3) secreting cell line WEHI‐3 supernatant. A549 was cultured in Ham's F‐12K (Kaighn's) Medium (GIBCO, Thermo Fisher Scientific) with 10% FBS. The medium was mixed with 100 units mL^−1^ penicillin and 100 µg mL^−1^ streptomycin. Cells were cultured in a humidified atmosphere containing 5% CO_2_ at 37 °C. Male nude mice (4–6 weeks) were obtained from Shanghai SLAC Laboratory Animals Co., Ltd. (Shanghai, China) and housed under pathogen‐free conditions. All animal experiments were approved by the Animal Care and Use Committee at the Shanghai Jiao Tong University.

*In Vitro Cell Viability Assays*: The cell viability assays were performed with CCK8 (DOJINDO, Japan) as previously described.^30^ Cells were treated with helicenium at 0–100 µg mL^−1^ for 24 h.

*Cell Apoptosis and Cell Cycle Assays*: Cells (2 × 10^5^ cells/well) were seeded in six‐well plates and cultured for 24 h. [4]Helicenium was added to the cells at a dose of 10 µg mL^−1^ for 24 h. For optical imaging, cells were directly observed and imaged under a light microscope. For apoptosis assay, the cells were trypsinized, washed with cold PBS, and resuspended in 100 µL of 1× binding buffer containing 5 µL Annexin V and 1 µL PI (Invitrogen) at room temperature for 15 min in the dark followed by adding 400 µL of 1× binding buffer to each tube. After the incubation, the perception of apoptotic cells was measured by FACSCalibur flow cytometry (BD Biosciences, USA) and analyzed with FlowJo Software. For cell cycle analysis, the cells were resuspended in 300 µL PBS and 700 µL of cold ethanol was dropped into the cells while shaking. The cells were fixed at −20 °C overnight. Then the cells were washed with PBS, stained with PI (500 µg mL^−1^) for 30 min, and immediately analyzed by flow cytometry.

*RNA Sequencing and q‐PCR*: For RNA sequencing, total RNA was extracted from SK‐Hep1 and HL7702 cells using RNeasy Mini Kit (QIAGEN, Cat. 74106) following the manufacturer's instructions. Qualified total RNA was further purified by RNAClean XP Kit (Beckman Coulter, Cat. A63987) and RNase‐Free DNase Set (QIAGEN, Cat. 79254). Then the purified total RNA was subjected to steps of mRNA isolation, fragmentation, first‐strand cDNA synthesis, second‐strand cDNA synthesis, end repair, 3′ end addition of A, linker, and enrichment to complete the library construction of the sequencing samples. cDNA samples were sequenced using a high‐throughput sequencer (Illumina Hiseq 2000/2500). The sequencing reads were mapped to the human genome (hg38) using Hisat2 (version: 2.0.4). Stringtie (version: 1.3.0) was used to calculate fragments per kilobase of exon model per million mapped reads (FPKM) values. Differential genes were analyzed by edgeR based on twofold changes. GO enrichment analyses were performed with DAVID software (http://david.abcc.ncifcrf.gov/). For q‐PCR, 1 µg of total RNA was reverse transcripted with the RT Reagent Kit with gDNA Eraser (TaKaRa). RT‐PCR was performed with the SYBR Premix Ex Taq Kit (TaKaRa). The relative gene expression levels were calculated using the ΔΔCt method, and GAPDH was used as the internal control. Primers will be provided upon request.

*Western Blot Analysis*: The treated cells were collected and lysed with 1× loading sample buffer (LI‐COR). After separation by sodium dodecyl sulfate polyacrylamide gel electrophoresis (SDS‐PAGE), the cell protein was transferred to NC membranes. The membranes were blocked by 5% BSA for 1 h, followed by an overnight incubation at 4 °C with anti‐P53 (Santa Cruz, Cat. sc‐126), anti‐P21 (Cell Signaling Technology, CST, Cat. 2947), anti‐Phospho‐CDC2 (CST, Cat. 4539), anti‐CDC2 (CST, Cat. 9116), anti‐β‐actin (Hangzhou HuaAn Biotechnology Co., Ltd., China, Cat. R1207‐1), anti‐FANCA (abcam, Cat. ab201458), anti‐phospho‐histone H2AX (CST, Cat. 2577), anti‐histone H3 (abcam, Cat. ab1791), and anti‐RAD51 (CST, Cat. 8875) antibodies, respectively. After incubation with the secondary antibodies labeled with fluorescence (LI‐COR, Cat. 926‐68070, Cat. 926‐68071), the proteins were visualized by fluorescence with an Infrared Imaging System (ODYSSEY). β‐actin and H3 were used as the internal control.

*Mouse Model*: 1 × 10^7^ SK‐Hep1 cells were injected subcutaneously into the dorsal flank of the four‐week‐old male BALB/c‐nude mice. Mice were randomly divided into four groups with an average of tumor volume of 150 mm^3^ (*n* = 40).

*In Vivo Drug Treatment*: The four groups of mice were injected intravenously 200 µL of normal saline, BQ, NQ, and [4]helicenium (3 mg kg^−1^), respectively. All the mice were treated every 2 d for four weeks. The body weight and tumor volume of each mouse were recorded every 4 d. At the end of the treatment, the peripheral blood serum of all mice was taken to detect biochemical indicators, and the vital organs were taken for HE staining.

*Gene Set Enrichment Analysis*: Normalized RNA‐seq data were rank‐ordered by fold change. GSEA analyses were performed using gene sets from the Molecular Signatures Database (MsigDB, hallmark gene sets and curated gene sets) with GESA v2.2.3 software.

*Immunofluorescence Staining*: The treated cells were fixed with 4% paraformaldehyde for 10 min, followed by 0.1% Triton X‐100 for 10 min at room temperature. Cells were incubated at 4 °C overnight with rabbit anti‐MCM2 (CST, Cat. 3619) and anti‐phospho‐histone H2AX antibodies, then incubated with Alexa Fluor 488 goat anti‐rabbit IgG[H+L] antibody (Invitrogen) for 1 h. DAPI staining was conducted subsequently. The images were examined and captured by a fluorescence microscope (Leica, DMI3000B, Germany).

*Single‐Cell Gel Electrophoresis (SCGE, the Comet Assay)*: DNA damage was detected using the adaption of the method of Singh et al.^31^ with some modification. Briefly, SK‐Hep1 and HL7702 cells (2 × 10^5^ cells/well) were cultured in six‐well dishes and were treated with or without 10 µg mL^−1^ [4]helicenium for 24 h. Then the cells were trypsinized, centrifuged, counted, and mixed with 0.5% low‐melting‐point agarose in PBS at 37 °C. The mixture was immediately pipetted onto a frosted glass microscope slide precoated with a layer of 0.5% normal‐melting‐point agarose in PBS. The agarose was placed at 4 °C for 10 min and the slide was immersed in 4 °C lysis solution for 1 h to dissolve the cell membranes and remove proteins and RNA. The slide was then washed twice by PBS and immersed in the freshly prepared alkaline solution for 20 min followed by electrophoresis in 4 °C alkaline electrophoresis solution at 25 V for 30 min. The slide was gently drained by filter paper to remove excess electrophoresis and then immersed in 0.4 m Tris‐HCl, pH 7.5 for 15 min before staining with 50 µL ethidium bromide (30 µg mL^−1^). The stained slide was viewed under a fluorescent microscope. The DNA damage was measured as tail length and tail moment.

*Statistical Analysis*: All data were expressed as the mean ± SD based on at least three independent experiments. Student's unpaired two‐tailed *t*‐test was used for analyzing the statistical significance of differences, with 0.05 selected as the significance level. The data were indicated with (*) for a probability less than (*p* < 0.05), (**) for *p* < 0.01, (***) for *p* < 0.001, and (****) for *p* < 0.0001, respectively. All statistical analyses were performed with GraphPad Prism 6.0 software (GraphPad Software, San Diego, CA).

## Conflict of Interest

The authors declare no conflict of interest.

## Supporting information

Supplementary
