# Supplementary material for: Selective Killing of Cancer Cells by Nonplanar Aromatic Hydrocarbon‐Induced DNA Damage
Source: Adv Sci (Weinh). 2019 Sep 16;6(21):1901341. doi: 10.1002/advs.201901341 (PMC6839640; doi:10.1002/advs.201901341)
Supplement: Supplementary file 1 — Supplementary [file ADVS-6-1901341-s001.pdf]

Copyright WILEY-VCH Verlag GmbH & Co. KGaA, 69469 Weinheim, Germany, 2019.

ADVANCED SCIENCE
Open Access

Supporting Information

for *Adv. Sci.,* DOI: 10.1002/advs.201901341

Selective Killing of Cancer Cells by Nonplanar Aromatic Hydrocarbon-Induced DNA Damage

Yan Zhou, Fuwei Gan, Yuanliang Zhang, Xiaozhen He, Chengshuo Shen, Huibin Qiu, and Peifeng Liu**

Copyright WILEY-VCH Verlag GmbH & Co. KGaA, 69469 Weinheim, Germany, 2019.

Supporting Information

Selective Killing of Cancer Cells by Nonplanar Aromatic Hydrocarbon-Induced DNA Damage

*Yan Zhou, Fuwei Gan, Yuanliang Zhang, Xiaozhen He, Chengshuo Shen, Huibin Qiu, *and Peifeng Liu**

Experimental Section

Synthesis of PAHs:

(1) Synthesis of [4]helicenium

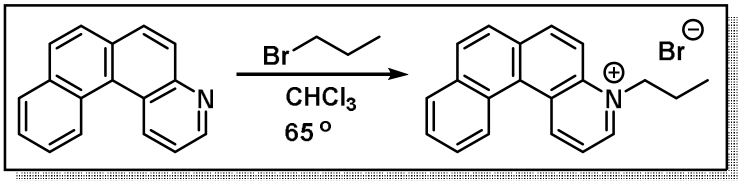

Scheme S1. Synthetic route for [4]helicenium.

115 mg of 4-aza[4]helicene (0.5 mmol) was suspended in a mixed solution with 3 mL $CHCl_3$ and 1.5 mL 1-bromopropane. The mixture was stirred at 65 °C for 48h. Yellow solid was produced as the reaction going. Then the solvent was removed by vacuum. The solid was dissolved in CH_2Cl_2 and was precipitated by adding Et_2O. The solid was collected by centrifugation. The process of dissolution-precipitation-centrifugation was repeated for three times. Then the solid was kept in a vacuum oven to remove residual solvent. The product was obtained as the yellow solid (123 mg, yield 70%).

^{1}H NMR (500 MHz, DMSO-*d*6, 295 K): δ 10.10 (d, J = 8.6 Hz, 1H), 9.61 (d, J = 5.7 Hz, 1H), 8.89 (d, J = 8.2 Hz, 1H), 8.80 (d, J = 9.3 Hz, 1H), 8.75 (d, J = 9.3 Hz, 1H), 8.36 (d, J = 8.2 Hz, 1H), 8.35-8.28 (m, 2H), 8.24 (d, J = 8.6 Hz, 1H), 7.95-7.86 (m, 2H), 5.19 (t, J = 7.6 Hz, 2H), 2.09 (m, 2H), 1.04 (t, J = 7.3 Hz, 3H). ^{13}C NMR (125 MHz, DMSO-*d*6, 295 K): δ

146.89, 145.69, 138.92, 137.05, 133.91, 130.96, 130.80, 129.15, 128.33, 128.28, 128.08, 127.82, 127.37, 126.05, 126.03, 122.14, 116.99, 59.41, 23.27, 10.61.

(2) Synthesis of Quinolinium (Q)

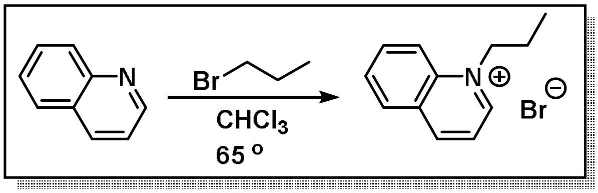

Scheme S2. Synthetic route for Q.

129 mg of quinoline (1 mmol) was suspended in a mixed solution with 3 mL $CHCl_3$ and 1.5 mL 1-bromopropane. The mixture was stirred at 65 °C for 48h. Yellow solid was produced as the reaction going. Then the solvent was removed by vacuum. The solid was dissolved in CH_2Cl_2 and was precipitated by adding Et_2O. The solid was collected by centrifugation. The process of dissolution-precipitation-centrifugation was repeated for three times. Then the solid was kept in a vacuum oven to remove residual solvent. The product was obtained as the yellow solid (152 mg, yield 60%).

^{1}H NMR (500 MHz, DMSO-*d*6, 295 K): δ 9.62 (d, J = 5.6 Hz, 1H), 9.32 (d, J = 8.3 Hz, 1H), 8.66 (d, J = 9.0 Hz, 1H), 8.51 (d, J = 8.2 Hz, 1H), 8.28 (t, J = 7.9 Hz, 1H), 8.21 (dd, J = 8.3, 5.8 Hz, 1H), 8.06 (t, J = 7.6 Hz, 1H), 5.06 (t, J = 7.6 Hz, 2H), 2.00 (m, 2H), 0.97 (t, J = 7.4 Hz, 3H). ^{13}C NMR (125 MHz, DMSO-*d*6, 295 K): δ 149.64, 147.39, 137.40, 135.60, 130.72, 129.86, 129.70, 122.09, 118.98.

(3) Synthesis of Benzo[*f*]quinolinium (BQ)

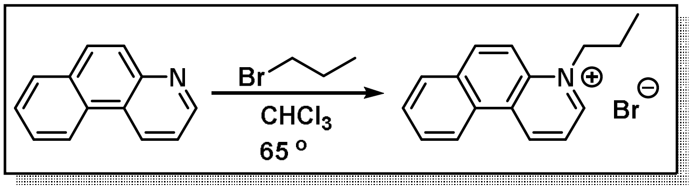

Scheme S3. Synthetic route for BQ.

179 mg of benzo[*f*]quinoline (1 mmol) was suspended in a mixed solution with 3 mL $CHCl_3$ and 1.5 mL 1-bromopropane. The mixture was stirred at 65 °C for 48h. Yellow solid was produced as the reaction going. Then the solvent was removed by vacuum. The solid was dissolved in CH_2Cl_2 and was precipitated by adding Et_2O. The solid was collected by centrifugation. The process of dissolution-precipitation-centrifugation was repeated for three times. Then the solid was kept in a vacuum oven to remove residual solvent. The product was obtained as the yellow solid (160 mg, yield 53%).

^{1}H NMR (500 MHz, DMSO-*d*6, 295 K): δ 10.15 (d, J = 8.6 Hz, 1H), 9.57 (d, J = 6.9 Hz, 1H), 9.13 (d, J = 8.2 Hz, 1H), 8.70 (d, J = 9.6 Hz, 1H), 8.55 (d, J = 9.6 Hz, 1H), 8.36 (dd, J = 8.6, 5.8 Hz, 1H), 8.34 (d, J = 5.8 Hz, 1H), 8.02 (t, J = 6.9 Hz, 1H), 7.97 (t, J = 6.9 Hz, 1H), 5.12 (t, J = 7.6 Hz, 2H), 2.04 (m, 2H), 1.00 (t, J = 7.3 Hz, 3H). ^{13}C NMR (125 MHz, DMSO-*d*6, 295 K): δ 147.17, 141.41, 138.74, 137.79, 130.87, 129.97, 129.97, 129.41, 128.34, 128.16, 124.20, 122.77, 116.14, 59.10, 23.24, 10.51.

(4) Synthesis of Naphtho[2,1-*f*]quinolinium (NQ)

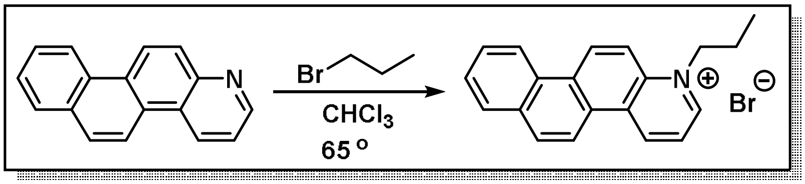

Scheme S4. Synthetic route for NQ.

115 mg of naphtho[2,1-*f*]quinoline (0.5 mmol) was suspended in a mixed solution with 3 mL $CHCl_3$ and 1.5 mL 1-bromopropane. The mixture was stirred at 65 °C for 48h. Yellow solid was produced as the reaction going. Then the solvent was removed by vacuum. The solid was dissolved in CH_2Cl_2 and was precipitated by adding Et_2O. The solid was collected by centrifugation. The process of dissolution-precipitation-centrifugation was repeated for three times. Then the solid was kept in a vacuum oven to remove residual solvent. The product was obtained as the yellow solid (92 mg, yield 52%).

^{1}H NMR (500 MHz, DMSO-*d*6, 295 K): δ 10.26 (d, J = 8.7 Hz, 1H), 9.64 (d, J = 9.8 Hz, 1H), 9.61 (d, J = 5.3 Hz, 1H), 9.14 (d, J = 8.3 Hz, 1H), 9.10 (d, J = 9.2 Hz, 1H), 8.75 (d, J = 9.8 Hz, 1H), 8.44 (d, J = 9.2 Hz, 1H), 8.39 (dd, J = 8.6, 5.8 Hz, 1H), 8.26 (d, J = 7.9 Hz, 1H), 7.92 (t, J = 7.6 Hz, 1H), 7.87 (t, J = 7.4 Hz, 1H), 5.19 (t, J = 7.4 Hz, 2H), 2.03-2.15 (m, 2H), 1.03 (t, J = 7.3 Hz, 3H). ^{13}C NMR (125 MHz, DMSO-*d*6, 295 K): δ 147.77, 142.43, 137.94, 132.47, 132.11, 130.90, 129.03, 128.82, 128.55, 128.32, 128.31, 128.04, 127.54, 123.95, 122.58, 121.06, 116.64, 59.06, 23.22, 10.57.

Liquid Chromatography-Tandem Mass Spectrometry (LC-MS) analysis: For detecting the intracellular and extracellular concentration of [4]helicenium, all cell supernatant was aspirated and the cells were collected after treatment with 10 μg mL^{-1} [4]helicenium. 300 μL of the supernatant was added to 900 μL of pre-cooled methanol, mixed and placed at −80 °C for 0.5 h. After centrifugation at 13,200 rpm for 20 min at 4 °C, the supernatant was directly detected. 1 mL of pre-cooled methanol was added to the cells, thawed with liquid nitrogen for 5 times and then ultrasonicated three times for 30 s each time. After centrifugation at 13,200 rpm for 20 min at 4 °C, the supernatant was dried with nitrogen and then resuspended in 600 μL of 50% methanol for LC-MS analysis. LC separation was using a SHIMADZU Nexera LC-30A UHPLC system and Kinetex C18 column (50 mm × 2.1 mm, 2.6 μm; Phenomenex). Mobile phase A was water with 0.1% formic acid. Mobile phase B was 90: 10 acetonitrile: water with 0.1% formic acid. The injection volume was 3 μL and LC gradient conditions were: 0 min: 20% B; 2.5 min: 90% B; 3.5 min: 90% B; 3.9 min: 20% B; 6 min: 20% B, flow rate was 0.4 mL min^{-1} and column temperature was 40 °C. MS detection was using an SCIEX 6500 QTRAP mass spectrometer with ESI source operating in MRM (272.1/230.1) and positive ionization mode. MS parameters were: temperature: 400 °C; Collision Energy: 35 V; spray voltage: 5000 V. Quantitation was performed using calibration curve and the Mutiquant software (Sciex). For metabolic stability detection, human and liver microsomes were used as in vitro models. [4]Helicenium at a concentration of 3 μM was

incubated with microsomal protein and coenzyme NADPH, and the reaction was stopped by adding ice-cold acetonitrile. The $T_{1/2}$ was determined by the incubation time and the In value of the drug residual rate in the incubation system, and then the intrinsic clearance (CL_{int}) and the liver clearance (CL_{hb}) value were calculated. For detecting the distribution of [4]helicenium in vivo, the main organs and plasma of four SD rats intravenously injected with 2 mg kg^{-1} [4]helicenium for 1 h and 8 h were collected. After homogenization of the tissues with a high-speed homogenizer, the concentration of [4]helicenium in plasma, whole blood and tissue homogenate were determined by LC-MS/MS. For detecting the absorption of [4]helicenium in vivo, the concentration of [4]helicenium in plasma was measured by LC-MS/MS at different times after intravenous and intragastric administration in rats at 2 mg kg^{-1} and 5 mg kg^{-1}, respectively.

Supplementary figures

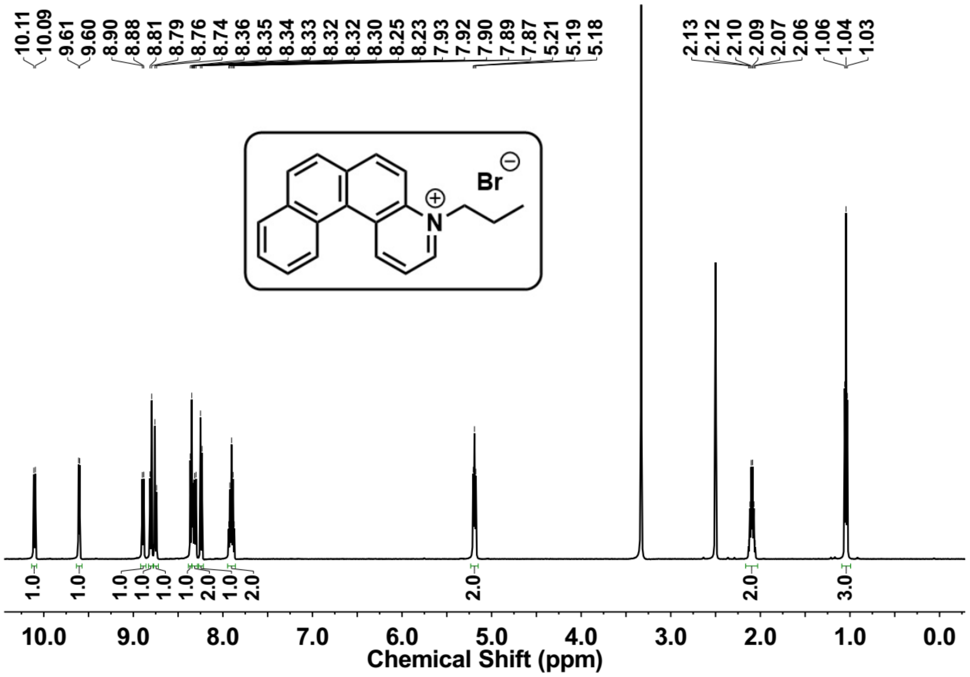

Figure S1. ^{1}H NMR spectrum of [4]helicenium (500 MHz, DMSO-*d*6, 295 K).

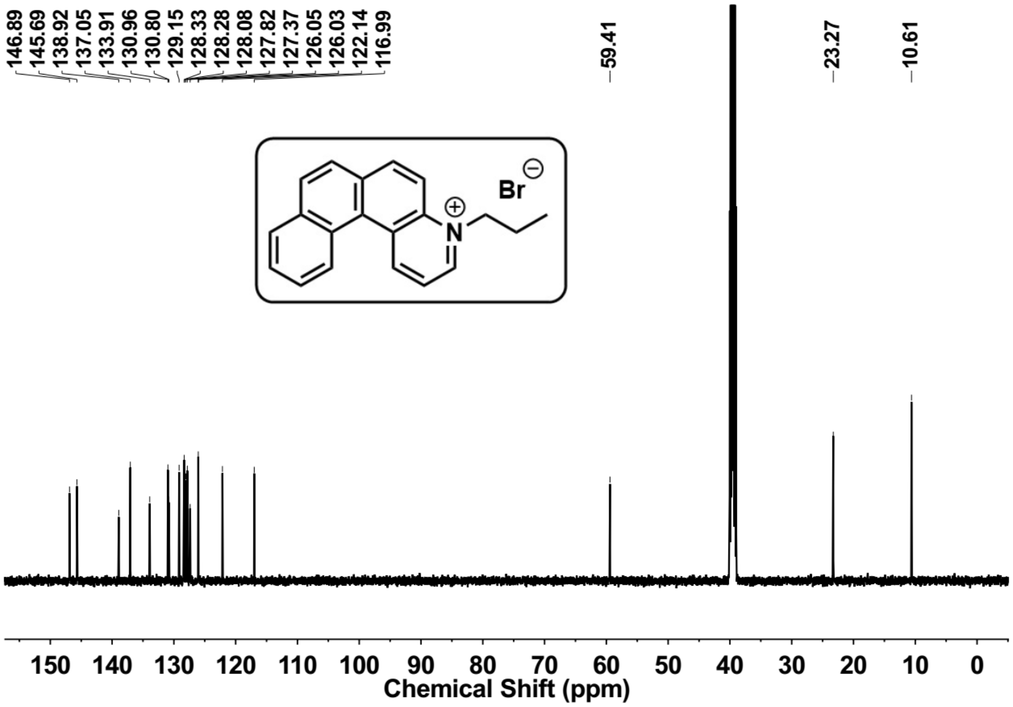

Figure S2. ^{13}C NMR spectrum of [4]helicenium (125 MHz, DMSO-*d*6, 295 K).

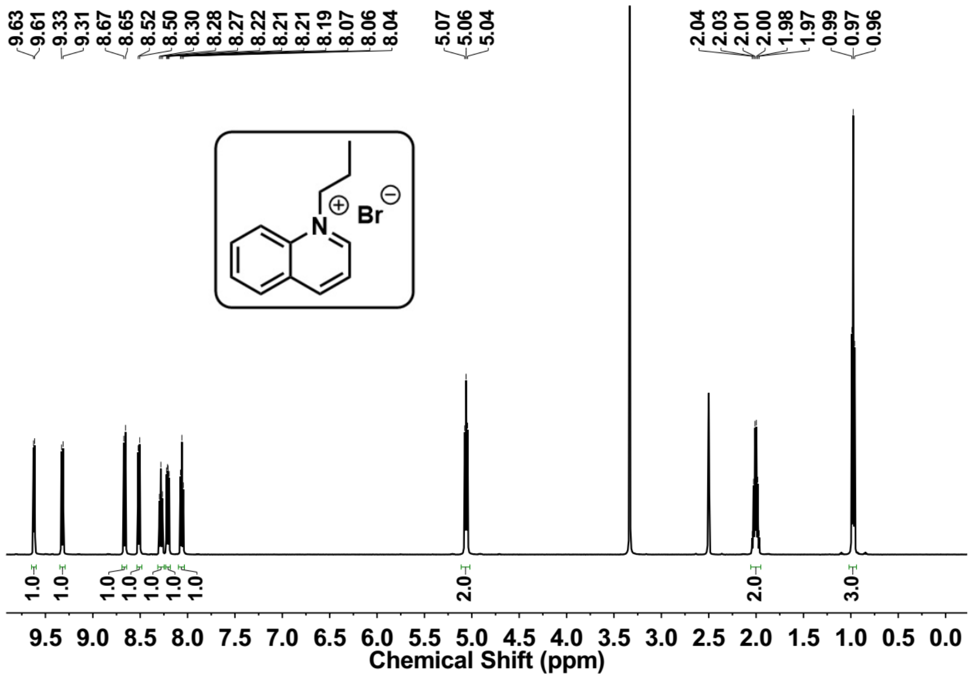

Figure S3. ^{1}H NMR spectrum of Q (500 MHz, DMSO-*d*6, 295 K).

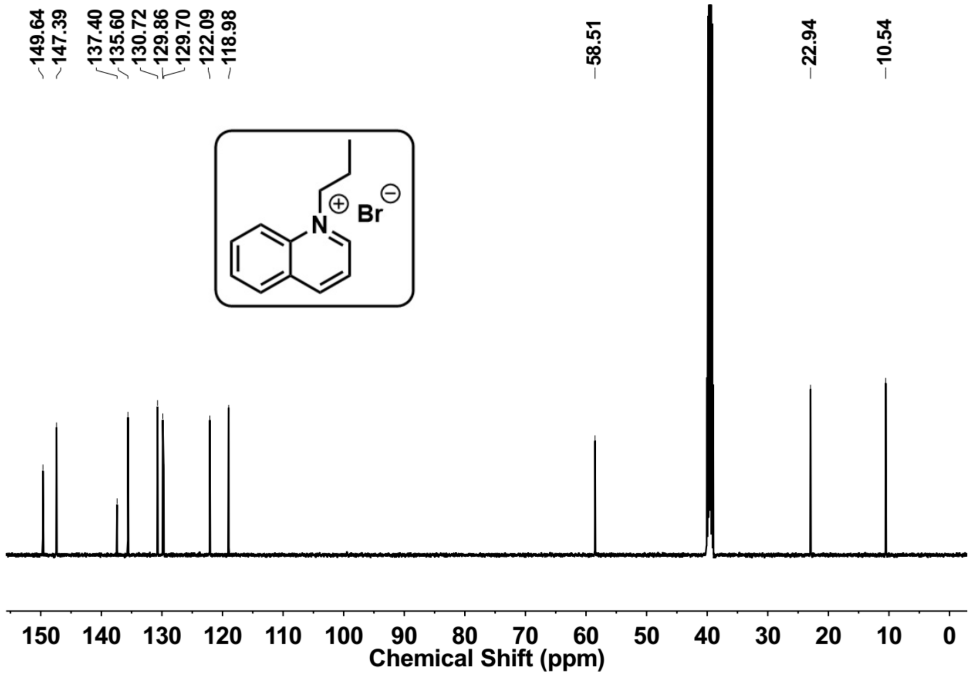

Figure S4. ^{13}C NMR spectrum of Q (125 MHz, DMSO-*d*6, 295 K).

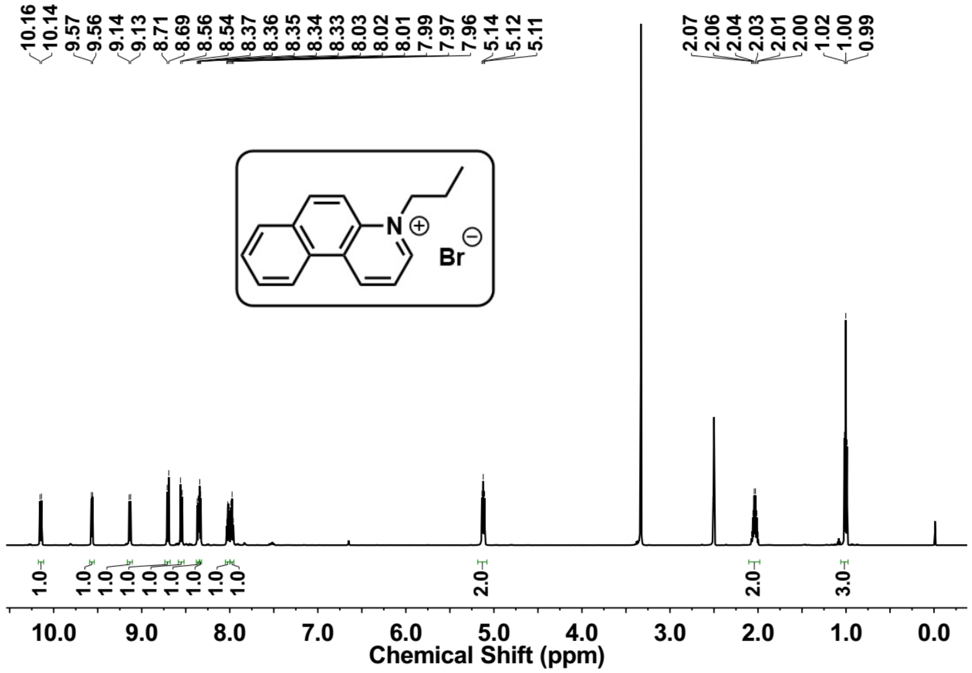

Figure S5. ^{1}H NMR spectrum of BQ (500 MHz, DMSO-*d*6, 295 K).

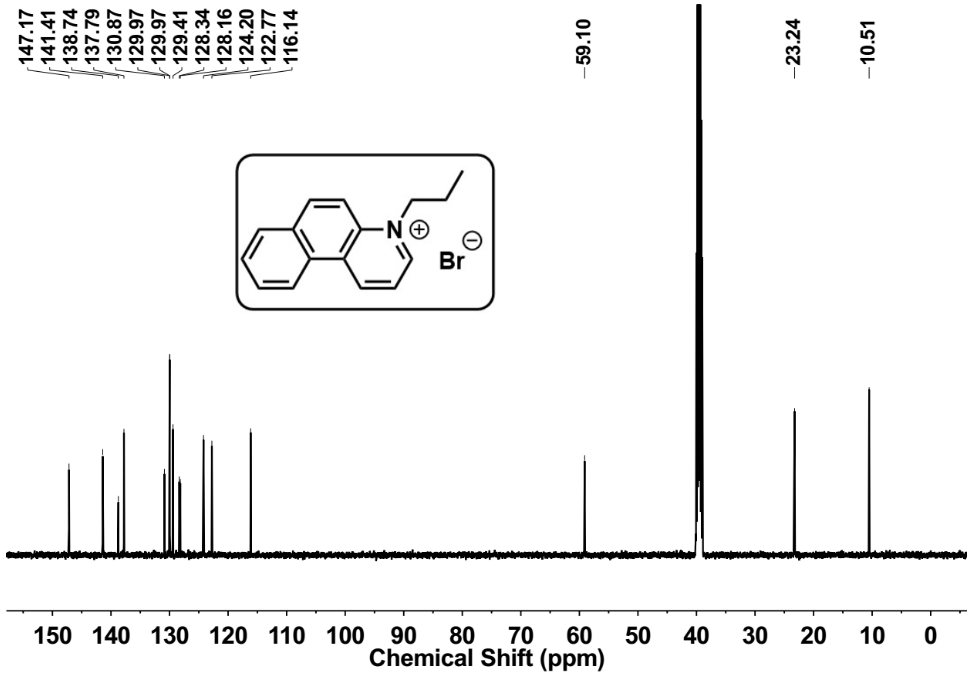

Figure S6. ^{13}C NMR spectrum of BQ (125 MHz, DMSO-*d*6, 295 K).

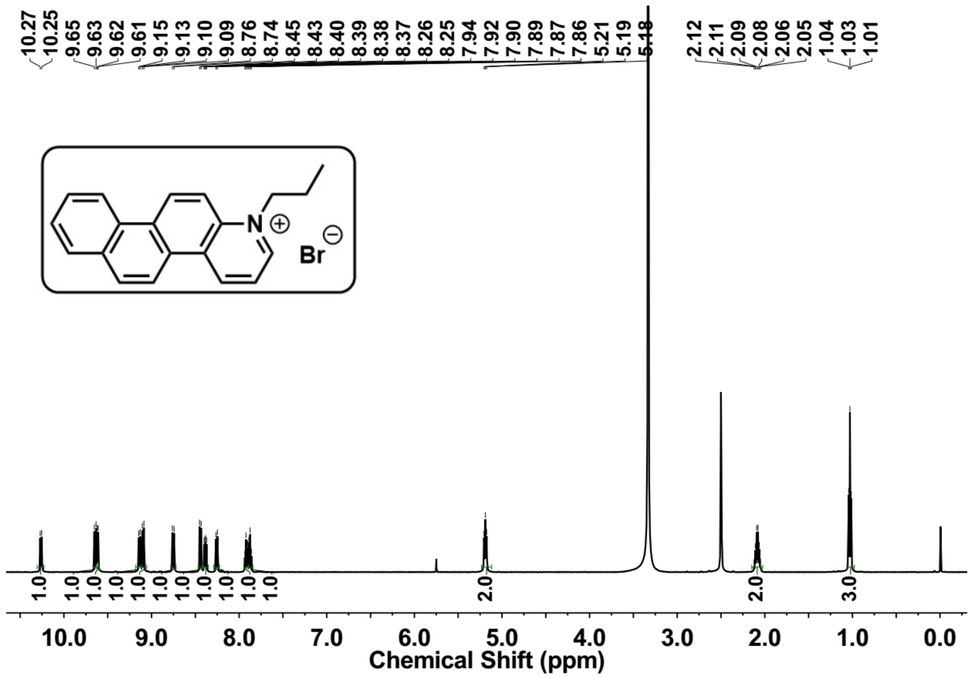

Figure S7. ^{1}H NMR spectrum of NQ (500 MHz, DMSO-*d*6, 295 K).

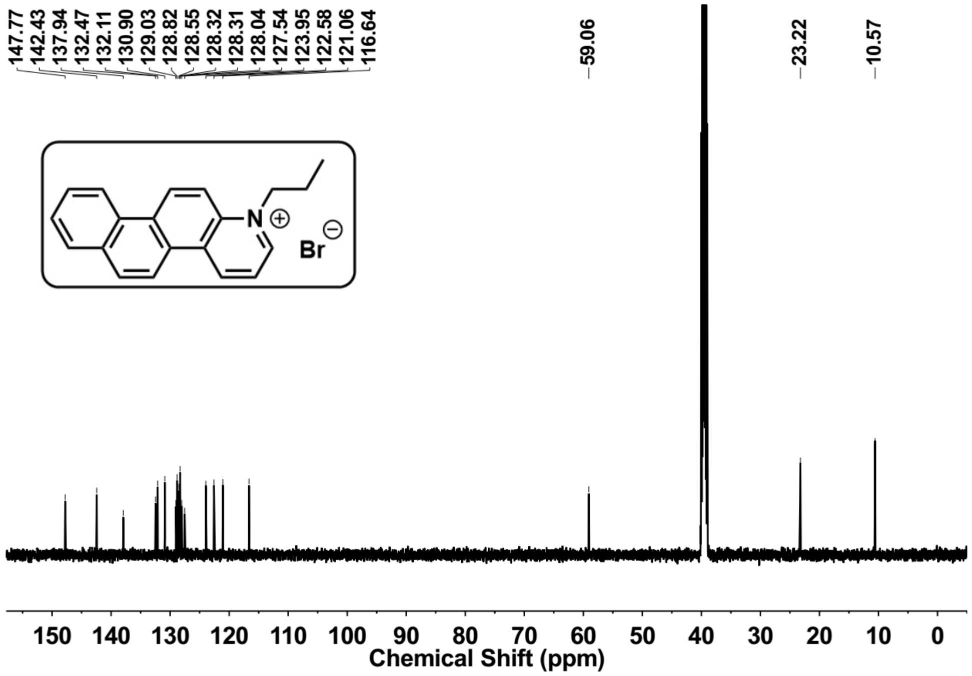

Figure S8. ^{13}C NMR spectrum of NQ (125 MHz, DMSO-*d*6, 295 K).

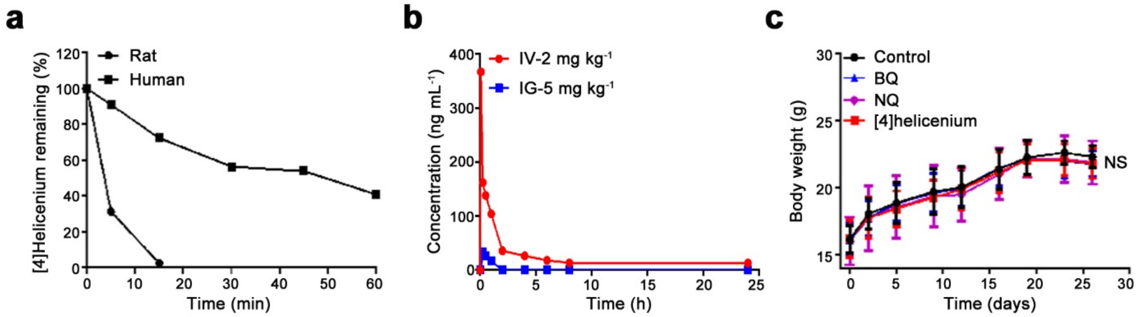

Figure S9. a) Clearance rates of [4]helicenium in human and rat liver microsomes in vitro. b) Mean plasma concentration-time curve after intravenous and intragastric administration of [4]helicenium in rats. IV: intravenous, IG: intragastric. c) Body weight profiles of healthy nude mice treated with normal saline, BQ, NQ and [4]helicenium.

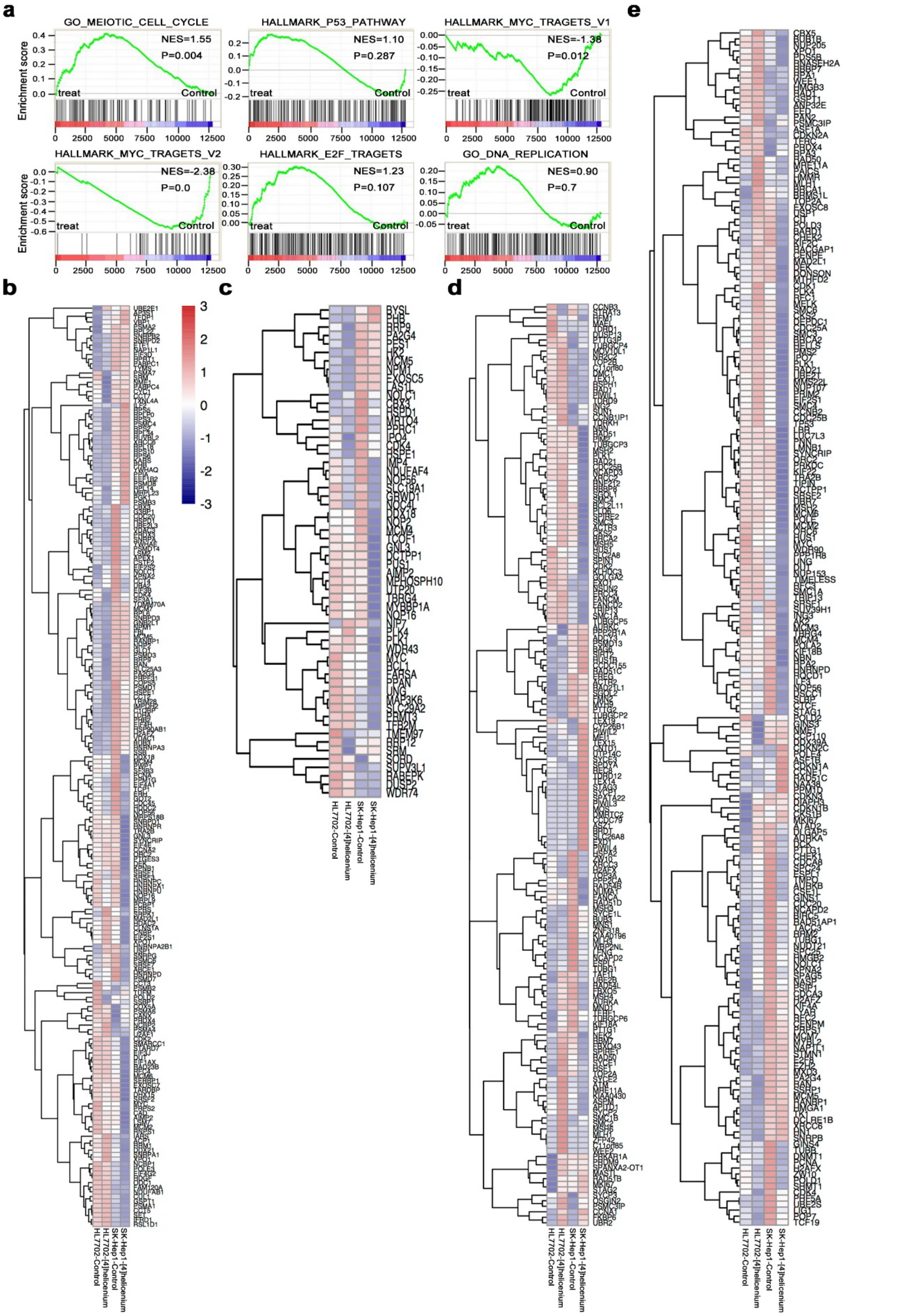

Figure S10. a) GSEA of the entire transcriptome of HL7702 before and after [4]helicenium treatment. b-e) Heatmap analysis of individual gene expression in gene-sets of MYC targets V1 (b), MYC targets V2 (c), meiotic cell cycle (d) and E2F targets (e).

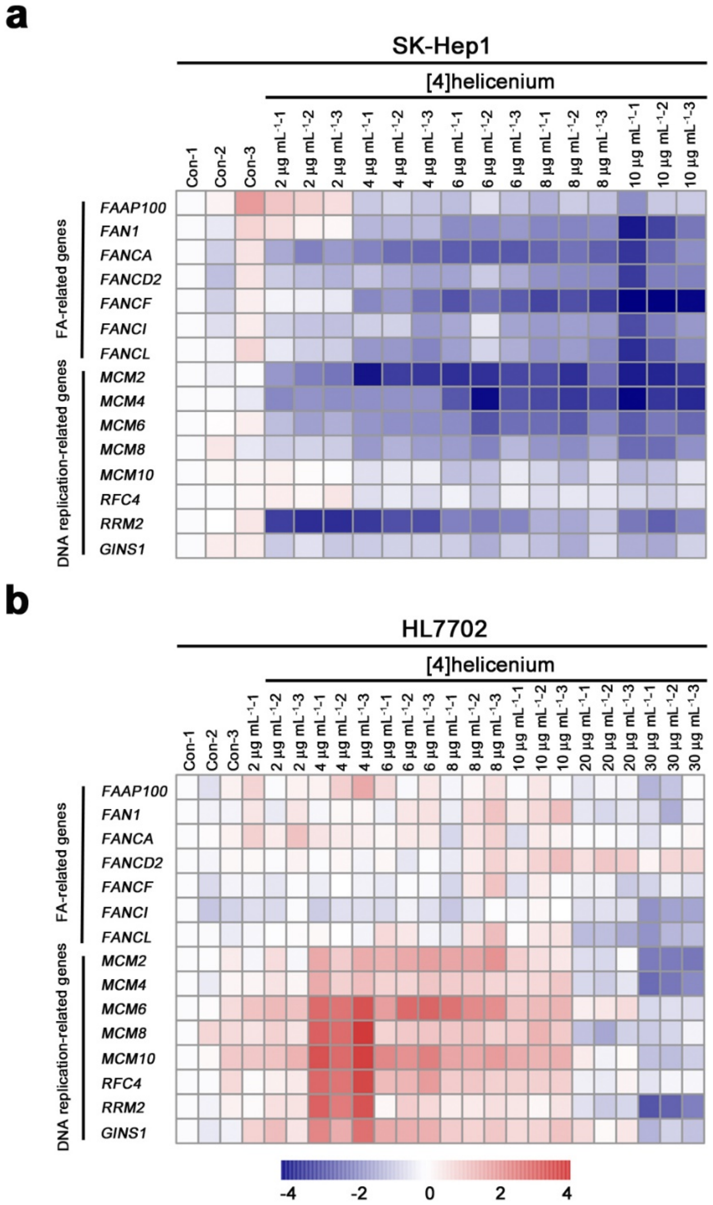

Figure S11. Heatmap of the expression levels of FA- and DNA replication-related genes detected by q-PCR in SK-Hep1 (a) and HL7702 (b) treated with different concentrations of [4]helicenium.

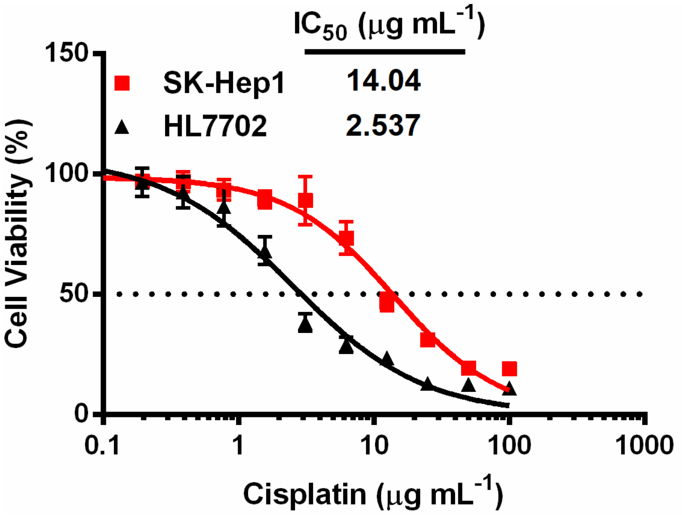

Figure S12. Cell viability analysis of SK-Hep1 and HL7702 treated with cisplatin at 0~100 μg mL^{-1} for 24 h.

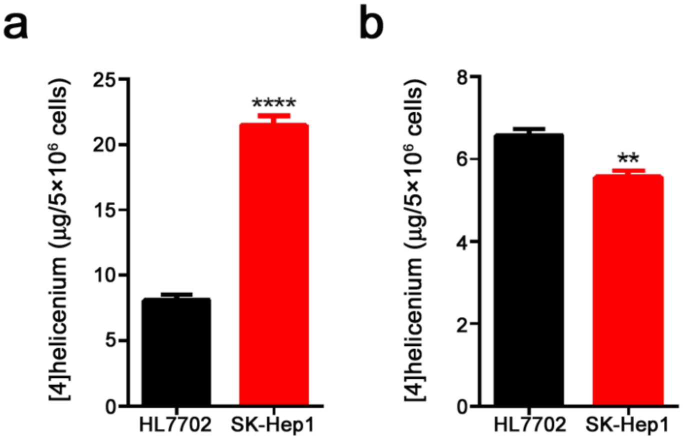

Figure S13. The content of [4]helicenium in HL7702 and SK-Hep1 cells (a) and cell supernatant (b) detected by liquid chromatograph-tandem mass spectrometry.

tissue	1h				8h			
	Rat1 (ng g^{-1})	Rat2 (ng g^{-1})	Average (ng g^{-1})	ratio/plasma (ng mL^{-1})	Rat3 (ng g^{-1})	Rat4 (ng g^{-1})	Average (ng g^{-1})	ratio/plasma (ng mL^{-1})
heart	10900	10150	10525	152.6	15350	12950	14150	626.1
kidney	8550	11950	10250	148.7	165	192	178	7.9
pancreas	2455	2095	2275	33.0	1465	1430	1448	64.0
womb	1675		1675	24.3	221		221	9.8
small intestine	1250	1700	1475	21.4	305	319	312	13.8
lung	1300	1190	1245	18.1	153	126	140	6.2
stumch	1150	1275	1213	17.6	223	156	189	8.4
overy	1110		1110	16.1	202		202	8.9
bladder	730	770	750	10.9	295	282	289	12.8
skin	595	600	598	8.7	204	267	235	10.4
spleen	550	403	476	6.9	119	104	111	4.9
testis		168	168	2.4		62.5	62.5	2.8
blood	118	85.8	102	1.5	35.7	24.1	29.9	1.3
plasma	92.4	45.5	69.0	1.0	16.9	28.3	22.6	1.0
liver	43.0	40.3	41.6	0.6	BQL	BQL	0	0.0
brain	26.0	BQL	26.0	0.4	BQL	BQL	0	0.0

Table S1. Distribution of [4]helicenium in rat tissues and plasma at 1h and 8h after treatment with 2 mg kg^{-1} [4]helicenium.
